# Development of a Real-Time Human-Robot Collaborative System Based on 1 kHz Visual Feedback Control and Its Application to a Peg-in-Hole Task †

**DOI:** 10.3390/s21020663

**Published:** 2021-01-19

**Authors:** Yuji Yamakawa, Yutaro Matsui, Masatoshi Ishikawa

**Affiliations:** 1 Interfaculty Initiative in Information Studies, The University of Tokyo, Tokyo 153-8505, Japan; 2 Graduate School of Information Science and Technology, The University of Tokyo, Tokyo 113-8656, Japan; utaro0201@gmail.com; 3 Information Technology Center, The University of Tokyo, Tokyo 113-8656, Japan; ishikawa@ishikawa-vision.org

**Keywords:** Human-Robot collaboration, high-speed robot, high-speed vision, visual feedback

## Abstract

In this research, we focused on Human-Robot collaboration. There were two goals: (1) to develop and evaluate a real-time Human-Robot collaborative system, and (2) to achieve concrete tasks such as collaborative peg-in-hole using the developed system. We proposed an algorithm for visual sensing and robot hand control to perform collaborative motion, and we analyzed the stability of the collaborative system and a so-called collaborative error caused by image processing and latency. We achieved collaborative motion using this developed system and evaluated the collaborative error on the basis of the analysis results. Moreover, we aimed to realize a collaborative peg-in-hole task that required a system with high speed and high accuracy. To achieve this goal, we analyzed the conditions required for performing the collaborative peg-in-hole task from the viewpoints of geometric, force and posture conditions. Finally, in this work, we show the experimental results and data of the collaborative peg-in-hole task, and we examine the effectiveness of our collaborative system.

## 1. Introduction

Recently, research into Human-Robot interaction (HRI) has been actively undertaken. HRI contributes not only to industrial applications (for example, cell production systems) but also to human living environments (so-called Quality of Life (QoL) improvements). HRI techniques can be divided into three kinds:

1. Collaboration and cooperation,
2. Communication, and
3. Support and assistance.

In the area of collaboration and cooperation, robots perform tasks together with workers; in communication, robots enter into dialogue with humans; and in support and assistance, robots assist workers in performing tasks or actions. In this research, we focus on collaboration and cooperation. Human-Robot collaboration is a fundamental element for humans and robots to work together. Moreover, this field involves physical interactions between humans and robots.

To date, a great deal of research has been conducted on Human-Robot collaborative systems and cooperation systems [1]. Zoss et al. classified collaborative systems between humans and robots [2]. Hayashibara et al. developed an assistive system for carrying a long object [3]. Yokoyama et al. performed a task in which an object was held and carried by a Human-Robot cooperative system [4]. Kosuge et al. proposed a control algorithm of a mobile robot with dual arms for Human-Robot cooperation [5]. Suda and Kosuge constructed a system for handling objects using visual and force information [6]. Stückler and Behnke developed a system that followed human guidance to carry a large object during cooperation between a human and a robot [7]. Antao et al. proposed a method in which a manipulator assisted a human operator to execute a target task, while monitoring the operator in real-time [8]. Teke et al. proposed a method for real-time and robust collaborative robot motion control by using Kinect^®^ v2 [9]. Çoban and Gelen achieved an assembly task with Human-Robot collaboration using a wearable device and shortened the operation time of the assembly task [10]. Wang et al. proposed a framework of a TLC (teaching–learning–collaboration) model for performing Human-Robot collaborative tasks and verified the effectiveness of the proposed method [11]. Shayganfar et al. explored the relevance and controllability of Human-Robot collaboration and suggested an evaluation algorithm for relevance and controllability [12]. Scimmi et al. achieved a hand-over task with a robot manipulator by using real-time visual information [13]. Galin. et al. constructed a mathematical model and simulation environment of Human-Robot collaboration [14]. Darvish et al. proposed a hierarchical architecture of a Human-Robot cooperative system, showed the algorithms used to evaluate the architecture and performed experiments [15].

Considering HRI performance in the same way as robot performance, the speed and accuracy of the system can be considered to be the most important factors. The previous research described above focused on the accuracy and construction of HRI systems, including algorithms for improving accuracy. Currently, more-accurate HRI systems using advanced techniques and AI techniques have been proposed [1,16,17,18]; these studies focus on robot perception with gesture [16], the proposal of integrated frameworks [17] and support techniques in HRI with machine learning [18]. This means that speed (real-time performance) has not been considered in detail in these systems. As a result, HRI systems cannot react to human motion and actions instantly, and the human has to adapt to the slow robot motion and action. We consider that such a style is not ideal for HRI systems, and that real-time performance is critically important.

**Figure 1:**
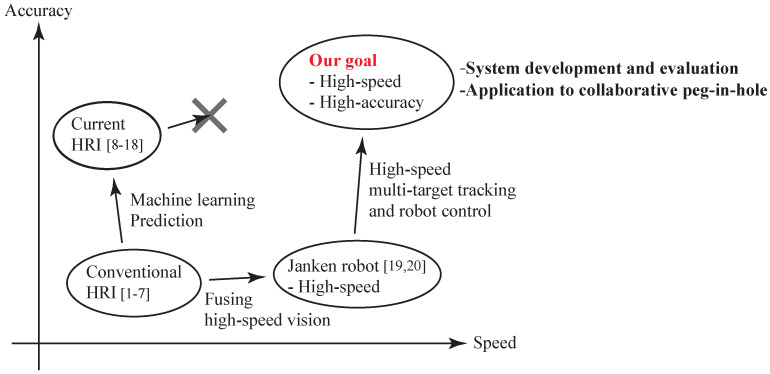
Goal of this research. HRI: Human-Robot interaction.

Thus, this research pursues the goal of a high-speed, high-accuracy HRI system, and the target area is the top-right area shown in Figure 1. To achieve this, we developed a high-speed, high-accuracy HRI system using high-speed vision (1000 fps image acquisition), high-speed image processing (1000 fps image processing) and high-speed robot control (real-time visual feedback). At present, the main approach that has been used to develop HRI systems is to improve the accuracy first by using machine learning and prediction, and then also improve the speed by speeding up these processing steps. With this approach, the authors consider that it is extremely difficult to speed up the processing. As a result, speed improvement cannot be achieved successfully. On the other hand, our approach is that the speed will be improved first by fusing high-speed vision and a high-speed robot, and then the accuracy will be also improved by using high-speed multi-target tracking and robot hand control. Based on this approach, we can achieve a high-speed (real-time performance) and high-accuracy HRI system. Such an approach can be considered to be a novel feature of this research.

**Figure 2:**
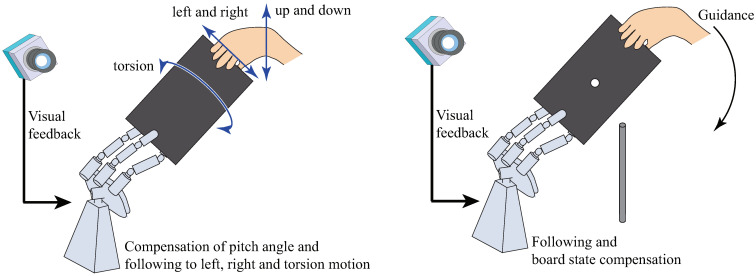
Purpose of this research [24].

In work related to the HRI system using a high-speed robot, we have also developed a Janken (rock–paper–scissors) robot with a 100% winning rate by using a high-speed robot [19,20]. From the results of that study, we considered that high-speed robot technology can be applied to Human-Robot collaboration. As a basic task, we decided on collaborative motion between a human and a robot hand, as shown in Figure 2. First, we constructed a simple Human-Robot collaborative system consisting of a high-speed robot hand and a high-speed vision system; this system holds an object (a board) horizontally via Human-Robot collaboration [21]. Second, we extended that system to a collaborative system that keeps the board horizontal, as in the previous research [21], but with the added function that the robot follows movements around the roll axis and yaw axis, performed by a human [22,23]. However, the analysis of the developed Human-Robot collaborative system was not performed. Thus, in this work, we analyze the system from theoretical and experimental aspects. Since the performance of a Human-Robot collaboration system is considered to depend on the error arising from image processing and the latency caused by a low frame rate (which we call the collaborative error), we analyze the stability of this Human-Robot collaborative system and evaluate this error through analysis and experiments [24]. In addition, we analyze the conditions of the achievement of the collaborative peg-in-hole task, and we demonstrate the collaborative peg-in-hole task using the developed collaborative system.

In this paper, we examine the following seven aspects:

1. The construction of a high-speed and high-accuracy Human-Robot collaborative system,
2. The proposed strategy for high-speed visual sensing and robot control in a high-speed and high-accuracy Human-Robot collaborative system,
3. The stability analysis of the high-speed and high-accuracy Human-Robot collaborative system,
4. The theoretical analysis of the collaborative error due to image processing and latency,
5. The experimental evaluation of the collaborative error and control performance (torque inputs) of the robot hand,
6. The analysis of the peg-in-hole task performed by the collaborative system, and
7. Te demonstration of a concrete application (peg-in-hole task) via Human-Robot collaboration.

The first and second aspects are related to the development of a new Human-Robot collaborative system, including an algorithm. The third and fourth aspects are related to the basic analysis of the Human-Robot collaborative system. The fifth aspect is related to the verification of the analysis results for the collaborative error through experiments. The sixth aspect is related to a basic analysis of the peg-in-hole task in the collaborative system. The last aspect is related to realization of the task using our high-speed and high-accuracy collaborative system. Through all seven of these aspects, we demonstrate the effectiveness of our high-speed Human-Robot collaborative system using a high-speed robot hand and high-speed image processing.

The rest of this paper is organized as follows; Section 2 explains the developed Human-Robot collaborative system, Section 3 describes the proposed strategy for Human-Robot collaboration, Section 4 discusses the stability and the collaborative error, Section 5 shows the experimental results of collaborative motion and discusses the frame rate of the collaborative system, Section 6 explains the analysis and experimental results of the collaborative peg-in-hole task and Section 7 summarizes the conclusions obtained in this work.

## 2. Human-Robot Collaborative System

**Figure 3:**
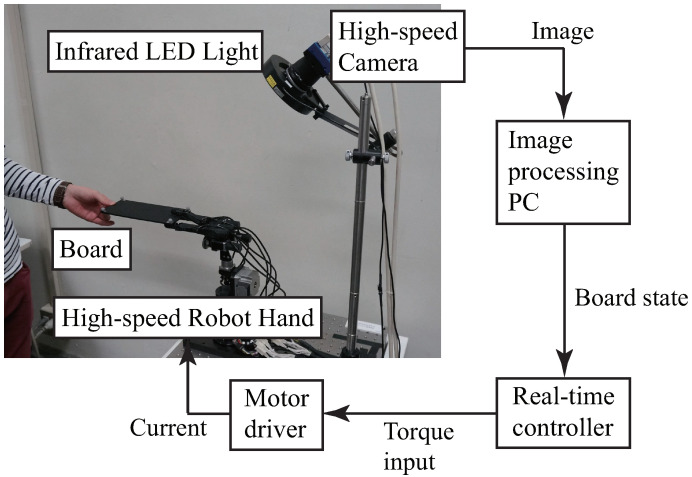
Human-Robot collaborative system [22].

As shown in Figure 3, our Human-Robot collaborative system consists of the following:

- A high-speed robot hand (Section 2.1),
- A high-speed vision system (Section 2.2),
- A real-time controller that receives the state values of the board (position and orientation) from the image-processing PC at 1 kHz and also controls the high-speed robot hand at 1 kHz,
- A board that is handled by the robot hand and a human subject (Section 2.3), and
- A peg (Section 2.4).

### 2.1. High-Speed Robot Hand

**Figure 4:**
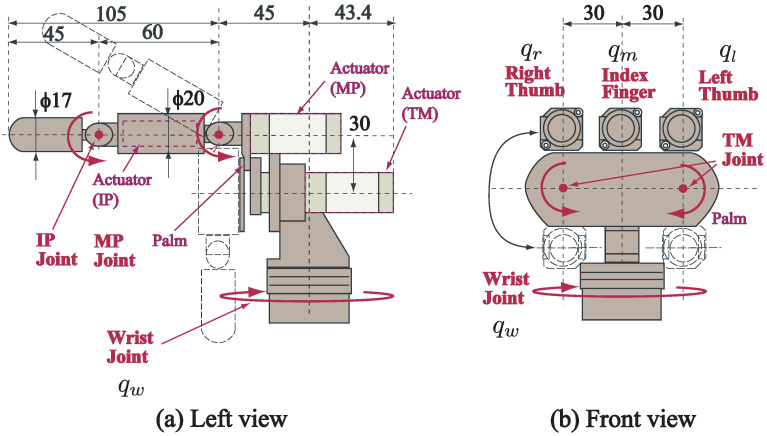
Mechanism of high-speed robot hand [25].

As the actuation system of the collaborative system, we used a high-speed robot hand, as shown in Figure 4 [25]. The joint of the robot hand has a closing speed of 180° in 0.1 s, which is a level of performance beyond that of a human hand.

The high-speed robot hand has three fingers: a left thumb, an index finger and a right thumb. Each finger has a top link and a root link, and the left and right thumbs rotate around a palm. Therefore, the index finger has two degrees of freedom (2-DOF), and both thumbs have 3-DOF. In addition, the robot hand has a wrist joint with 2-DOF (in Figure 4, 1-DOF movement is illustrated). Thus, the hand has a total of 10-DOF in its movement.

### 2.2. High-Speed Vision System

As the sensing system of the collaborative system, we used a high-speed vision system. The high-speed vision system consisted of a high-speed camera and an image-processing PC.

As the high-speed camera, we used a commercial high-speed camera, EoSens MC4086 produced by Mikrotron [26]. The image-processing PC was equipped with an Intel^®^ Xeon^®^ W5-1603 v3 2.8 GHz processor and 16 GB of RAM. The operating system of the image-processing PC was Windows 7 Professional (64-bit), and the image-processing software was Visual Studio 2017. The image-processing PC, equipped with a frame grabber board, could acquire raw image data from the high-speed camera. The connection between the high-speed camera and the image-processing PC used CoaXpress, which was able to transfer the data at high speed.

The raw image data were 1024 × 768 pixel, 8 bit gray-scale images. After acquiring the image data every 1 ms, the image-processing PC measured the position and orientation of the board within 1 ms and sent the measurement results to the real-time controller via an Ethernet connection using the UDP protocol.

### 2.3. Board with Hole as a Target Object

**Figure 5:**
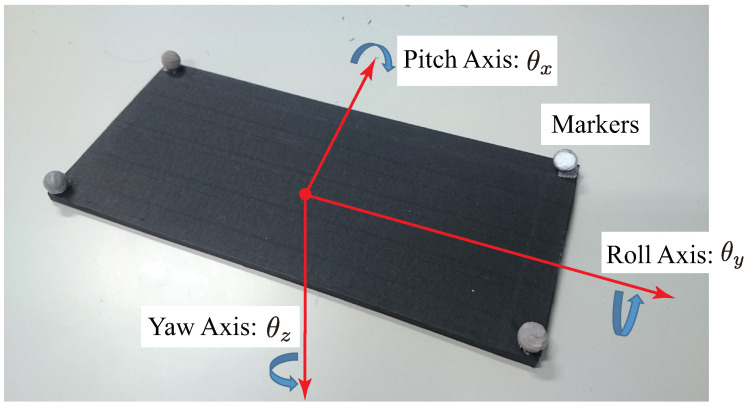
Configuration of axes on the board [22].

**Figure 6:**
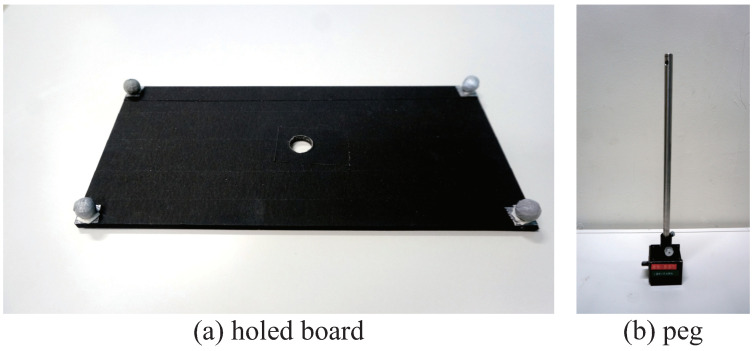
Board with hole and peg used in the collaborative peg-in-hole task.

The board had a length of 220 mm, a width of 100 mm, a thickness of 5 mm and a mass of about 113 g. Retro-reflective markers were attached at four corners of the board to simplify corner detection by the high-speed camera. The configuration of coordinate axes on the board was as shown in Figure 5. In addition, the hole used in the collaborative peg-in-hole task was formed at the center of the board, as shown in Figure 6a. The radius (*R*) of the hole was 6.350 mm.

### 2.4. Peg

The peg was made of stainless steel, had a radius (*r*) of 6.325 mm, a length ($L_{peg}$) of 405 mm, a chamfer angle (β) of 45° and a chamfer length (*w*) of 1 mm, as shown in Figure 6b. In general, the chamfer is formed around the edge of the hole; in this research, however, the chamfer was formed on the peg. In this case, the conditions for the peg-in-hole task were the same as those for the previous analysis result described in Section 4.1. The peg was fixed to the frame by a magnet.

## 3. Strategy for Collaborative Motion

**Figure 7:**
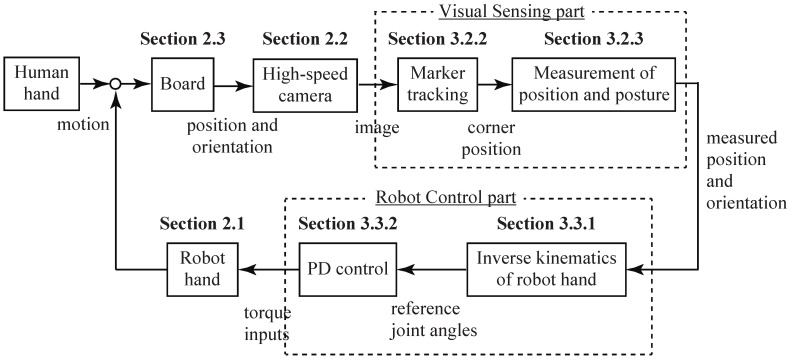
Control flow of Human-Robot collaborative system. PD: proportional derivative.

Here, we explain the overall strategy for achieving Human-Robot collaboration. The flow of the Human-Robot collaborative motion was the following, as shown in Figure 7:

1. The human subject moved the board,
2. The board position and orientation were changed as a result of the human operation,
3. The high-speed camera captured the image,
4. The tracking of the markers attached to the four corners of the board was executed by image-processing,
5. The position and posture of the board were calculated based on the information of the marker positions,
6. The reference joint angle of the robot hand was obtained by solving the inverse kinematics of the robot hand based on the position and posture of the board,
7. The torque to be input to the servo motor of the robot hand was generated by proportional derivative (PD) control for the reference joint angle, and
8. The robot hand moved according to the torque input.

### 3.1. Brief Overview

In the Human-Robot collaborative motion, we divided our strategy into the following two components:

1. Visual Sensing part in Figure 7: The position and orientation of the board were measured using high-speed image processing (Section 3.2).
2. Robot Control part in Figure 7: The robot hand was controlled according to the position and orientation of the board (Section 3.3).

By repeating the above two steps at high speed in real time (1000 fps), the board could be kept in the reference state, even if the human subject randomly moved the board at high speed. The visual sensing and robot control parts are explained below.

### 3.2. Image Processing and Measurement of Position and Orientation of Board

**Figure 8:**
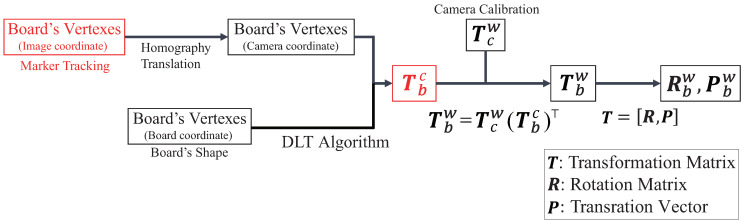
Flow of image processing and measurement of board state.

Figure 8 shows the flow of image processing and the measurement of the board state. In order to obtain the position and orientation of the board with global coordinates to control the robot hand, we needed to derive a transformation matrix $T_{b}^{w}$ from the board coordinates to global coordinates:

(1) $$T_{b}^{w}=\left[\begin{array}{cc} R_{b}^{w} & P_{b}^{w}\\ 0 & 1 \end{array}\right].$$

**Figure 9:**
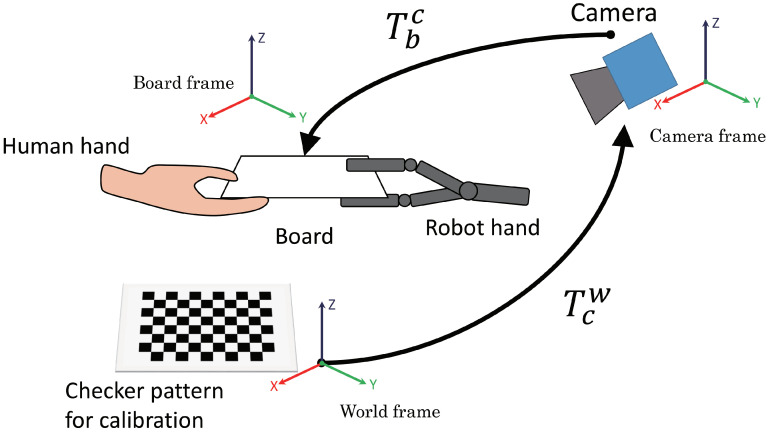
Relationship between transformation matrices T [22].

In Figure 8, the red elements are important for measuring the position and orientation of the board. Thus, we describe methods to calculate the transformation matrix $T_{b}^{w}$ (Section 3.2.1), to track the markers attached to the corners of the board visually (Section 3.2.2) and to convert the roll, pitch and yaw angles from the transformation matrix $T_{b}^{w}$ (Section 3.2.3). Figure 9 shows the relationship between transformation matrices.

#### 3.2.1. Derivation of Transformation Matrix

Assuming that a transformation matrix $T_{c}^{w}$ and a camera internal parameter matrix could be found in advance using camera calibration based on Zhang’s method [27], we were able to calculate a transformation matrix $T_{b}^{c}$. Therefore, we briefly describe how to obtain the transformation matrix $T_{b}^{c}$, which is composed of a rotation matrix $R_{b}^{c}$ and a translation vector $P_{b}^{c}$. If the transformation matrix $T_{b}^{c}$ is derived, the transformation matrix $T_{b}^{w}$ can be calculated as follows:

(2) $$T_{b}^{w}=T_{c}^{w}T_{b}^{c}\;.$$

Next, we describe a method of deriving the transformation matrix $T_{b}^{c}$.

We obtained the transformation matrix $T_{b}^{c}$ through the following calculation by using the direct linear transformation (DLT) algorithm. The transformation matrix $T_{b}^{c}$ could be expressed using the camera’s internal and external parameters as follows:

(3) $$s\left[\begin{array}{c} x\\ y\\ 1 \end{array}\right]=K\left[\begin{array}{cc} R_{b}^{c} & P_{b}^{c} \end{array}\right]\left[\begin{array}{c} X\\ Y\\ Z\\ 1 \end{array}\right]=\left[\begin{array}{ccc} f_{x} & 0 & c_{x}\\ 0 & f_{y} & c_{y}\\ 0 & 0 & 1 \end{array}\right]\left[\begin{array}{cc} R_{b}^{c} & P_{b}^{c} \end{array}\right]\left[\begin{array}{c} X\\ Y\\ Z\\ 1 \end{array}\right]$$

where *s* is a scaling factor, $f_{x}$ and $f_{y}$ are the focal lengths, $c_{x}$ and $c_{y}$ represent the centre of the image, [x,y] are image coordinates, [X,Y,Z] are world coordinates (here, board coordinates), $R_{b}^{c}$ is a rotation matrix from camera coordinates to board coordinates, and $P_{b}^{c}$ is a translation vector from camera coordinates to board coordinates. Here, the camera’s internal parameter K could be derived in advance by camera calibration using Zhang’s method [27]. Thus, we needed to derive the camera’s external parameters $R_{b}^{c}$ and $P_{b}^{c}$.

The transformation matrix $T_{b}^{c}$ can also be represented by

(4) $$\left[\begin{array}{c} x_{i}\\ y_{i}\\ 1 \end{array}\right]=H\left[\begin{array}{c} X_{i}\\ Y_{i}\\ Z_{i}\\ 1 \end{array}\right]\left(H={\left[\begin{array}{ccc} h_{1} & h_{2} & h_{3} \end{array}\right]}^{T}=\left[\begin{array}{ccc} f_{x} & 0 & c_{x}\\ 0 & f_{y} & c_{y}\\ 0 & 0 & 1 \end{array}\right]\left[\begin{array}{cc} R & P \end{array}\right]/s\right)\;.$$

The above equation can be rewritten using a vector representation as follows:

(5) $$x_{i}=\left[\begin{array}{c} h_{1}^{T}\\ h_{2}^{T}\\ h_{3}^{T} \end{array}\right]X_{i}\;.$$

Rewriting this equation for the vector $h_{i}$, we can get

(6) $$\left[\begin{array}{ccc} 0 & -X_{i}^{T} & y_{i}X_{i}^{T}\\ -X_{i} & 0 & -x_{i}X_{i}^{T} \end{array}\right]\left[\begin{array}{c} h_{1}\\ h_{2}\\ h_{3} \end{array}\right]=0\;.$$

A matrix A is defined by four components $x_{i},X_{i}$ (i=1,2,3,4) as follows:

(7) $$A=\left[\begin{array}{ccc} 0 & -X_{1}^{T} & y_{1}X_{1}^{T}\\ -X_{1}^{T} & 0 & -x_{1}X_{1}^{T}\\ 0 & -X_{2}^{T} & y_{2}X_{2}^{T}\\ -X_{2}^{T} & 0 & -x_{2}X_{2}^{T}\\ 0 & -X_{3}^{T} & y_{3}X_{3}^{T}\\ -X_{3}^{T} & 0 & -x_{3}X_{3}^{T}\\ 0 & -X_{4}^{T} & y_{4}X_{4}^{T}\\ -X_{4}^{T} & 0 & -x_{4}X_{4}^{T} \end{array}\right]$$

By performing singular value decomposition for the matrix A, we can get H. Then, we can also obtain $T_{b}^{c}$. Each component of the matrix A can be derived from the values $x_{i}$ and $X_{i}$ (i=1,2,3,4). The values $x_{i}$ can be obtained by the marker tracking, as described in the next session. The values $X_{i}$ (i=1,2,3,4) can also be determined from the board size.

#### 3.2.2. Marker Tracking

The positions of the four corners were obtained by using a target tracking algorithm [28]. By attaching retro-reflective markers to the four corners of the board and binarizing the captured image, the board appeared white only at the corners. By calculating the image moment for each marker, the positions of the four corners were obtained.

The marker tracking operation was performed as follows. First, the obtained image was binarized with a threshold. Second, the (i,j)-th order image moments $m_{i,j}$ were calculated by

(8) $$m_{i,j}=\sum_{x}\sum_{y}x^{i}y^{j}I\left(x,y\right)\;.$$

Using the image moments $m_{i,j}$, we were able to obtain the image centroid ($x_{g},y_{g}$) of the marker as follows:

(9) $$x_{g}=\frac{m_{1,0}}{m_{0,0}},y_{g}=\frac{m_{0,1}}{m_{0,0}}\;.$$

Once the marker image was captured and the centroid ($x_{g},y_{g}$) was successfully calculated, a sub-frame region of interest (ROI) was set around the centroid. The ROI size was set to be smaller than the size of the original image to reduce the computational load.

This tracking operation was executed for each marker, and the positions of the four corners could be obtained.

#### 3.2.3. Measurement of Board Position and Orientation in World Coordinates

From the transformation matrices $T_{c}^{w}$ and $T_{b}^{c}$, the transformation matrix $T_{b}^{w}$ from the board coordinates to the global coordinates was obtained. Consequently, the rotation matrix $R_{b}^{w}$ and the translation vector $P_{b}^{w}$ could be obtained; that is, the board position and orientation in global coordinates were measured.

The pitch, roll and yaw angles are expressed by $\theta_{x}$, $\theta_{y}$, $\theta_{z}$. The rotation matrix $R_{b}^{w}$ could be obtained from the transformation matrix $T_{b}^{w}$:

(10) $$R_{b}^{w}=\left[\begin{array}{ccc} r_{11} & r_{12} & r_{13}\\ r_{21} & r_{22} & r_{23}\\ r_{31} & r_{32} & r_{33} \end{array}\right]=R_{z}R_{x}R_{y}=\left[\begin{array}{ccc} c_{z}c_{y}-s_{z}s_{x}s_{y} & -s_{z}c_{x} & c_{z}s_{y}+s_{z}s_{x}c_{y}\\ s_{z}c_{y}+c_{z}s_{x}s_{y} & c_{z}c_{x} & s_{z}s_{y}-c_{z}s_{x}c_{y}\\ -c_{x}s_{y} & s_{x} & c_{x}c_{y} \end{array}\right]$$

As a result, the pitch, roll and yaw angles could be calculated as follows:

(11) $$\theta_{x}=\sin^{-1}\left(r_{32}\right),\;\theta_{y}=\tan^{-1}\left(\frac{-r_{31}}{r_{33}}\right),\;\theta_{z}=\tan^{-1}\left(\frac{-r_{12}}{r_{22}}\right)\;.$$

The series of image processing steps described above could be executed every 1 ms (1000 fps). The inverse kinematics of the robot hand were solved by a transformation matrix $T_{b}^{w}$ from the board coordinates to the world coordinates, and this is explained in the next subsection.

### 3.3. Robot Hand Control

In order to achieve the collaborative motion between the human and the robot, the robot hand was controlled based on the board position and orientation obtained as described above using the high-speed image processing. The robot hand control was also divided into two steps: solving the inverse kinematics of the robot hand and controlling the servo motors in the robot hand according to the reference joint angles.

#### 3.3.1. Inverse Kinematics of the Robot Hand

Using the inverse kinematics of the robot hand, the reference joint angles could be obtained based on the measurement of the board position and orientation. Moreover, since there is a limit to the range in which the robot hand could move, it was necessary to provide a limit to the input angles for moving the robot. Thus, we set an appropriate movable range for the board height.

**Figure 10:**
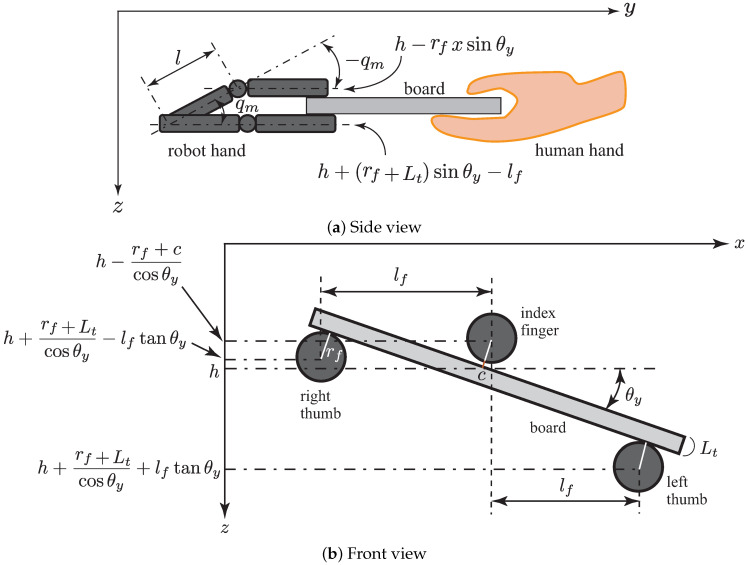
Inverse kinematics calculation of robot hand.

Figure 10 shows an illustration of the inverse kinematics of the robot hand. First, the height of the middle finger of the robot hand was derived based on the height of the center of the board:

(12) $$\left[\begin{array}{c} x\\ y\\ z=h\\ 1 \end{array}\right]=\left[\begin{array}{cccc} r_{11} & r_{12} & r_{13} & p_{x}\\ r_{21} & r_{22} & r_{23} & p_{y}\\ r_{31} & r_{32} & r_{33} & p_{z}\\ 0 & 0 & 0 & 1 \end{array}\right]\left[\begin{array}{c} 0\\ -l_{b}\\ 0\\ 1 \end{array}\right],$$

where $l_{b}$ is the distance from the center of the board to the edge grasped by the robot hand.

Then, the heights of the tip positions of the three fingers were derived as follows:

(13) $$z_{l}=h+\frac{r_{f}+L_{t}}{\cos\theta_{y}}+l_{f}\tan\theta_{y},\;z_{m}=h-\frac{r_{f}+c}{\cos\theta_{y}},\;z_{r}=h+\frac{r_{f}+L_{t}}{\cos\theta_{y}}-l_{f}\tan\theta_{y},$$

where $l_{f}$ is the distance between the fingers of the robot hand, $r_{f}$ is the radius of each finger, $L_{t}$ is the thickness of the board, and *c* is a small gap between the finger and the board. Therefore, the reference joint angles of the root links of the three fingers were calculated by

(14) $$q_{l}=\tan^{-1}\left(\frac{z_{l}}{l}\right),\;q_{m}=\tan^{-1}\left(\frac{z_{m}}{l}\right),\;q_{r}=\tan^{-1}\left(\frac{z_{r}}{l}\right)\;,$$

where *l* is the length of the root link of the finger. In order to set the top links parallel to the ground, the reference joint angles of the top links could be obtained by multiplying the reference joint angles of the root links by minus one. Moreover, the reference joint angle of the wrist was given by

(15) $$q_{w}=\theta_{z}\;.$$

The board was kept horizontal (the pitch angle $\theta_{x}=0$) by manipulating it, and so the top links had to be kept horizontal. Therefore, the joint angles of the top links were obtained by multiplying the joint angles of the root links by minus one.

#### 3.3.2. Joint Angle Control of the Robot Hand

In order to track the reference joint angles ($q_{l},q_{m},q_{r}$ and $q_{w}$) obtained by the inverse kinematics of the robot hand, the joint angles of the robot hand are controlled by proportional derivative (PD) control. Namely, the following torque input τ was applied to the actuator installed in the robot hand:

(16) $$\tau =k_{p}\left(\theta_{r}-\theta \right)+k_{d}\left({\dot{\theta }}_{r}-\dot{\theta }\right),$$

where $\theta_{r}$ is the reference joint angle of each joint of the robot hand and is calculated using Equation (14). θ is the actual joint angle of each joint of the robot hand and was measured by an optical encoder installed in the servo motor. Furthermore, $k_{p}$ and $k_{d}$ are the proportional and derivative gains, respectively.

### 3.4. Advantages and Limitations of Proposed Method

The advantages of the proposed methods are the high speed, low latency and high accuracy of the collaborative system. As a result, the system can collaborate with human motion in the true sense, which means that robot can react instantly and flexibly to human motion. In addition, in the conventional methods using a force sensor [29,30,31], it takes time for the sensor to measure the reaction force, to determine that the object was actually operated by the subject and to recognize the direction in which the object was moved. It can be considered that it is difficult to achieve these tasks and to speed up this approach. However, the proposed method is intuitive and can be recognized at high speed and with high accuracy.

On the other hand, the limitations of the proposed strategy are that it requires markers which are attached into four corners, and it also requires a lighting environment to detect the markers. At present, the target object is limited to plate-shaped objects, and additional ingenuity is required for visual sensing in order to adapt it to objects of other shapes. In addition, it is possible that strict camera calibration and system coordinate calibration are required for the realization of Human-Robot collaboration.

## 4. Theoretical Analysis

This section describes a theoretical analysis of the stability of the Human-Robot collaborative system and the theoretical collaborative error (particularly, pitch angle error $\theta_{x}$) that occurs during the collaborative motion.

### 4.1. Stability Analysis

The equations of the motion of the board (translational motion in the *z* direction and rotational motion around the pitch axis) during collaboration between a human and the robot system are given by

(17) $$\begin{array}{ccc} m\ddot{z} & = & mg-f_{r}-f_{h}\;, \end{array}$$

(18) $$\begin{array}{ccc} I\ddot{\phi } & = & mgL_{l}+m\ddot{z}L_{l}-2f_{r}L_{l}\;, \end{array}$$

where the moment of inertia of the board equals $I=\frac{1}{3}m{\left(2L_{l}\right)}^{2}$. In this analysis, we do not consider the torsional motion.

Next, we derive a force $f_{r}$ that acts on the board from the reference joint angles and the actual joint angles of the robot hand. Here, the reference joint angle $\theta_{ref}$ and the actual joint angle θ are as follows:

(19) $$\theta =\tan^{-1}\left(\frac{h+L_{l}\sin\phi }{l_{f}}\right),\;\theta_{ref}=\tan^{-1}\left(\frac{h-L_{l}\sin\phi }{l_{f}}\right)\;.$$

When the angle ϕ is small (ϕ≪1), the angles can be approximated as sinϕ≃ϕ and tanϕ≃ϕ. The force $f_{r}$ is equal to $\tau /(-L_{l})$ by using the approximation of the angles and the PD control law. Then, we can obtain

(20) $$f_{r}=2L_{l}\left(k_{p}\phi +k_{d}\dot{\phi }\right)\;.$$

Substituting $f_{r}$ into Equations (17) and (18), we can get

(21) $$\begin{array}{ccc} m\ddot{z} & = & mg-2\frac{L_{l}}{L_{f}^{2}}\left(k_{p}\phi +k_{d}\dot{\phi }\right)-f_{h}\;, \end{array}$$

(22) $$\begin{array}{ccc} I\ddot{\phi } & = & 2mgL_{l}-4\frac{L_{l}^{2}}{L_{f}^{2}}\left(k_{p}\phi +k_{d}\dot{\phi }\right)-f_{h}L_{l}\;. \end{array}$$

From these results, the transfer function G(s) from the force $f_{h}$ to the angle ϕ becomes

(23) $$G\left(s\right)=-\frac{3L_{f}^{2}/4L_{l}}{mL_{f}^{2}s^{2}+3k_{d}s+3k_{p}}\;.$$

In the case where there is no latency in the system, the transfer function G(s) is stable. However, the actual robot control system has some latency. Thus, assuming that the latency time is $T_{L}$, the transfer function $G_{L}\left(s\right)$ from a force $f_{h}$ to the angle ϕ can be rewritten as

(24) $$G_{L}\left(s\right)=-\frac{3L_{f}^{2}/4L_{l}}{mL_{f}^{2}s^{2}+3k_{d}s\frac{1}{1+T_{L}s}+3k_{p}\frac{1}{1+T_{L}s}}\;,$$

where the transfer function of the latency element is assumed to be a first-order lag system in order to allow modeling with a finite-dimensional function.

By applying Routh-Hurwitz stability analysis to the transfer function $G_{L}\left(s\right)$, the stability condition for the Human-Robot collaborative system is as follows:

(25) $$k_{d}-k_{p}T_{L}>0\;.$$

As a result, the stability condition for the latency time $T_{L}$ and the proportional and derivative gains $k_{p}$ and $k_{d}$ of the PD controller is

(26) $$T_{L}<\frac{k_{d}}{k_{p}}\;.$$

From this analysis result, we found that the latency time $T_{L}$ should be smaller than this value to stabilize the system. Additionally, the controller parameters could be adjusted from this condition under the determined sampling time $T_{L}$.

### 4.2. Analysis of Collaborative Error

This section explores the collaborative error due to the image-processing and the latency resulting from the frame rate. Here, we define the collaborative error as the pitch angle $\theta_{x}$. Namely, in the case where the pitch angle $\theta_{x}$ converges around 0 (this means that the board is kept horizontal), the collaborative error is considered to be small.

#### 4.2.1. Collaborative Error Due to Image-Processing

We evaluate the error due to the image-processing. Assuming that the projection error from camera calibration is $e_{r}$ pixel, the error of the image moment is $e_{p}$ pixel, the pixel size is *a* μm/pixel, the focal length is *f* mm and the distance between the camera and the board is $L_{c}$ mm, the measurement error of the corner position in the world coordinates can be calculated by

(27) $$e_{i}=\frac{aL_{c}}{f}\times \left(e_{r}+e_{p}\right)\times 10^{-3}\;\mathrm{mm}\;.$$

From the error $e_{i}$ obtained by Equation (27) and the board length $L_{l}$, the error $\theta_{pixel}$ due to the image-processing on the pitch-axis is given by

(28) $$\theta_{pixel}=\sin^{-1}\left(\frac{e_{i}}{L_{l}}\right)\;\mathrm{rad}\;.$$

From the experimental conditions shown in Table 1, we obtain $\theta_{pixel}=3.98\times 10^{-3}\;\mathrm{rad}$.

#### 4.2.2. Collaborative Error Due to Frame Rate

Second, we also evaluate the error resulting from the frame rate. Assuming that the board is moved by a human subject with an amplitude of *A* mm and frequency of $f_{freq}$ Hz, the board velocity is $2\pi Af_{freq}$ m/s. In the case where the frame rate is set at 1000 fps (1 ms), the latency becomes 3 ms, including 1 ms for image acquisition, 1 ms for image transmission and 1 ms for control. As a result, the error $\theta_{latency}$ due to the frame rate on the pitch-axis is also given by

(29) $$\theta_{latency}=\tan^{-1}\left(\frac{2\pi Af_{freq}\times \left(T_{m}+T_{t}+T_{c}\right)}{L_{l}}\right)\;\mathrm{rad}\;,$$

where $T_{m}$ is the measurement time for the image processing, $T_{t}$ is the transmission time of the data from the image processing PC to the real-time controller, and $T_{c}$ is the sampling time of the control. We assume that the transmission time and the sampling time are 1 millisecond each. $T_{m}$ depends on the frame rate of the high-speed camera. If the frame rate is 1000 fps, the measurement time becomes 1 millisecond. From the experimental conditions shown in Table 1, we obtain $\theta_{latency}=2.14\times 10^{-2}\;\mathrm{rad}$ in the case where the frame rate is 1000 fps.

The effect of the frame rate is about 10 times larger than the effect of the image processing. Consequently, the frame rate is very important for Human-Robot collaborative manipulation. We explored the validity of the collaborative errors in experiments that are described in the next section.

## 5. Experiment for Collaborative Motion Task

Finally, this section shows the experimental results of collaborative motion and the evaluation of the collaborative error and control performance due to the latency caused by the frame rate.

### 5.1. Result

**Figure 11:**
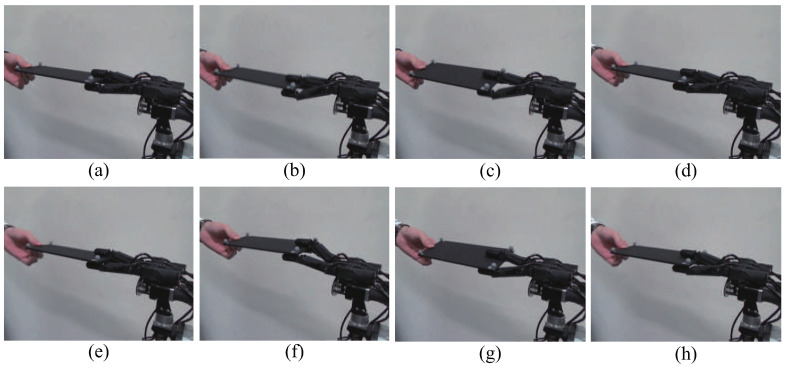
Sequential photographs of experimental results [22]. (**a**–**h**): the time interval of the sequential photographs is 1 s.

**Figure 12:**
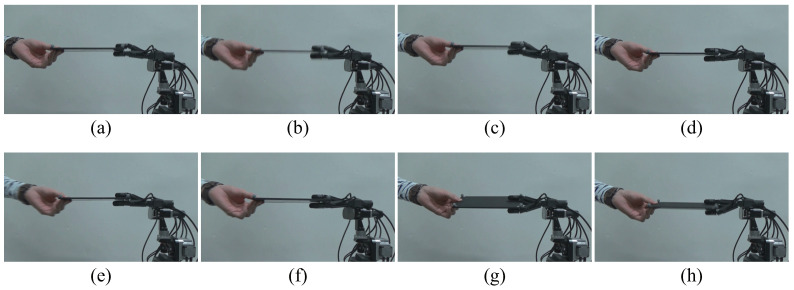
Sequential photographs of experimental results (side view). (**a**–**h**): the time interval of the sequential photographs is 0.5 s.

**Figure 13:**
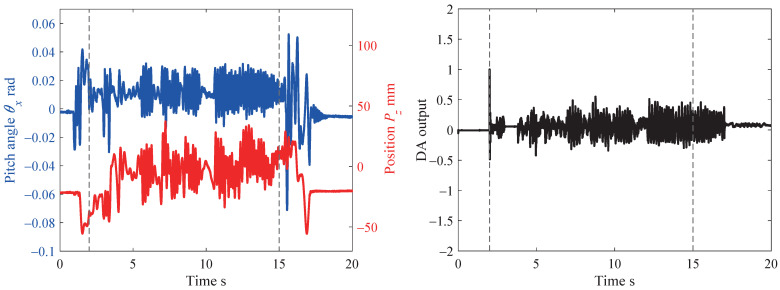
Data of experimental results.

Figure 11 and Figure 12 show continuous photographs of the experimental results at 1000 fps. The time intervals of the continuous photographs in Figure 11 and Figure 12 are 1 s and 0.5 s, respectively. Additionally, a video of the experimental results at 1000 fps is available on our website [32,33]. From the experimental results of the collaborative motion, the system was able to keep the board horizontal (the pitch angle $\theta_{x}$ was around zero) and followed the orientations with respect to the *y* and *z* axes. Figure 13 shows the collaborative error and DA output for the root link of the middle finger. The DA output corresponds to the torque input, and the limit for the DA output is set at ±1 (this means that the maximum torque input of each servo motor is generated). In the left side in Figure 13, the blue line and the red line depict the board angle $\theta_{x}$ and the board height $P_{z}$, respectively. In the right side in Figure 13, the black line shows the DA output.

In the experiment, the collaborative motion was performed in a time span from 2 to 15 s, which is depicted by the gray dotted line in Figure 13. It can be seen from Figure 13 that the collaborative error could be successfully suppressed to within 0.03 rad (≈1.8°) even when the board was moved by the human subject at a high speed and randomly. Furthermore, the torque input could be suppressed to within 0.5.

As a result, collaborative motion between the human and the robot hand using the developed system and proposed method was achieved.

### 5.2. Evaluation

**Figure 14:**
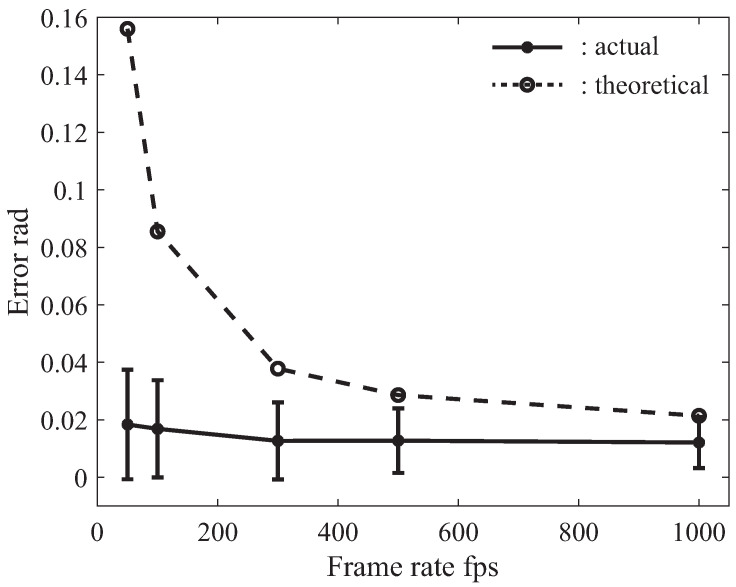
Theoretical collaborative error and actual collaborative error with various frame rates.

**Figure 15:**
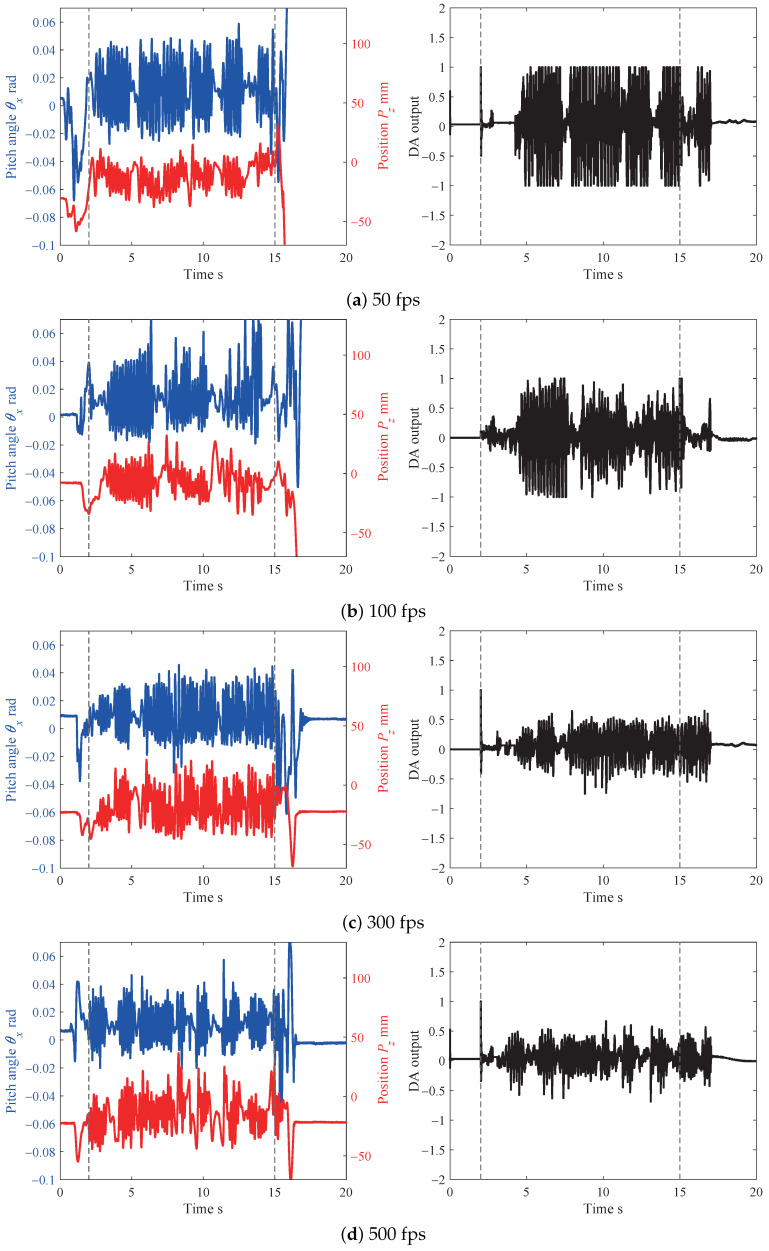
Comparison between various frame rates.

From the results described in Section 4.2, it can be seen that the collaborative error mainly depended on the frame rate. Therefore, we performed experiments with various frame rates: 50, 100, 300, 500 and 1000 fps. Figure 14 shows the theoretical and actual collaborative errors (pitch angle $\theta_{x}$), and Figure 15 shows the collaborative errors (left figures) and DA output (right figures) with various frame rates.

As shown in Figure 14, the theoretical collaborative error can be calculated by Equation (29), and the actual collaborative error can also be derived from the experimental result shown in Figure 15. In the plot of the actual collaborative error, the vertical bar depicts the standard deviation of the collaborative error. Theoretically, the lower the frame rate, the greater the collaborative error. In the experiments, on the other hand, when the frame rate decreased, the collaborative error did not increase significantly. This reason for this was that the responsiveness of the collaborative system was not good enough to realize collaborative motion, and the human subject unknowingly restricted and slowed down the board’s operation. However, the standard deviation increased slightly.

From Figure 15, we confirmed that even if the frame rate became low, collaborative motion could be achieved. However, the amplitudes of the collaborative error and DA output increased when the frame rate decreased. In particular, the difference in the DA output for the various frame rates was significant. In cases of low frame rates such as 50 and 100 fps, the DA output reached ±1. This means that the maximum signal to the servo motor was generated. On the other hand, the DA output was suppressed to be less than ±0.5 in case of high frame rates. As a result, we found that the Human-Robot collaborative system became stable and the load on the robot hand was reduced when the frame rate was high.

From the experimental results shown in Figure 13, Figure 14 and Figure 15, we confirmed that the pitch angle $\theta_{x}$ decreased when the frame rate increased. By increasing the frame rate (to over 300 fps), the stability of the Human-Robot collaborative system was improved, and the oscillation of the joint angles of the robot hand was also suppressed.

Next, we explain the analysis and experiment for the collaborative peg-in-hole task as a concrete task, which required high-accuracy performance as well as high-speed performance.

## 6. Collaborative Peg-In-Hole Task

In this section, we analyze the collaborative peg-in-hole task and clarify the conditions for achieving the task.

### 6.1. Conditions for Achieving Collaborative Peg-In-Hole Task

The peg-in-hole task has been widely investigated, and its modeling has also been studied. In the modeling, the condition described by Whitney has been analyzed and formulated using a peg-in-hole physical model [34,35]. In this section, we describe conditions for achieving the collaborative peg-in-hole task using our developed Human-Robot collaborative system, based on the conditions proposed by Whitney [34]. As a prior condition for the analysis, we assume that the human subject grasps one edge and the robot hand grasps one edge of the board.

#### 6.1.1. Geometric Conditions

**Figure 16:**
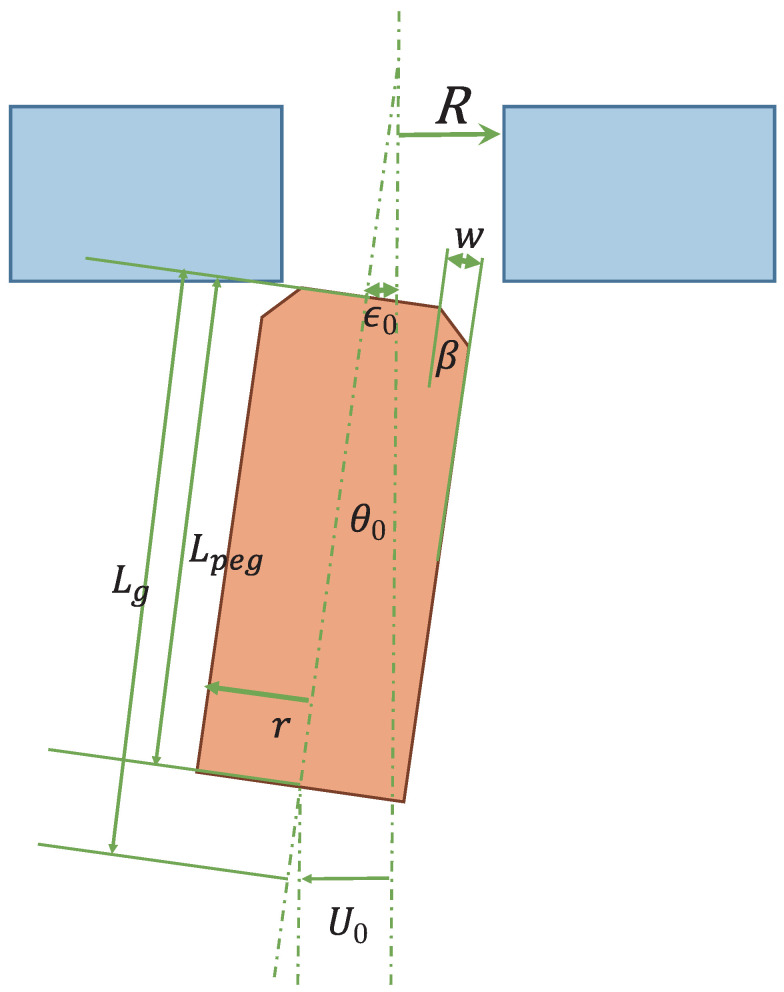
Peg shape and hole.

First of all, we consider geometric conditions for the peg-in-hole task. As a first condition, it was required that the peg and hole positions were adjusted to allow the peg to be inserted into the hole, as shown in Figure 16. As a permissible position error $e_{i}^{\prime }$, the condition ($e_{i}<e_{i}^{\prime }$) has to be satisfied, where $e_{i}$ is the actual position error, and $e_{i}^{\prime }$ can be calculated as follows:

(30) $$\left|e_{i}^{\prime }\right|\leq w\cos\theta_{0}+cR\leq w+cR\;\;\;\;\left(c=\frac{R-r}{R}\right)$$

In addition to the position adjustment, we also consider a condition for the board posture. As a permissible orientation error $\theta_{m}$, the condition $\theta^{\prime }<\theta_{m}$ has to be satisfied, where $\theta^{\prime }$ is the actual orientation error, and $\theta_{m}$ can be calculated as follows:

(31) $$\begin{array}{c} \theta_{m}=\cos^{-1}\left(\frac{r}{R}\right)\;, \end{array}$$

where $\theta^{\prime }=\theta_{pixel}^{\prime }+\theta_{latency}^{\prime }$. $\theta_{pixel}^{\prime }$ and $\theta_{latency}^{\prime }$ are collaborative errors due to the image processing and the latency, which can be calculated by Equations (28) and (29), respectively. Although the operation speed of the human subject was limited due to $\theta_{latency}^{\prime }$, the collaborative peg-in-hole task could be achieved when the condition $\theta_{pixel}^{\prime }<\theta_{m}$ was satisfied.

#### 6.1.2. Force Condition

**Figure 17:**
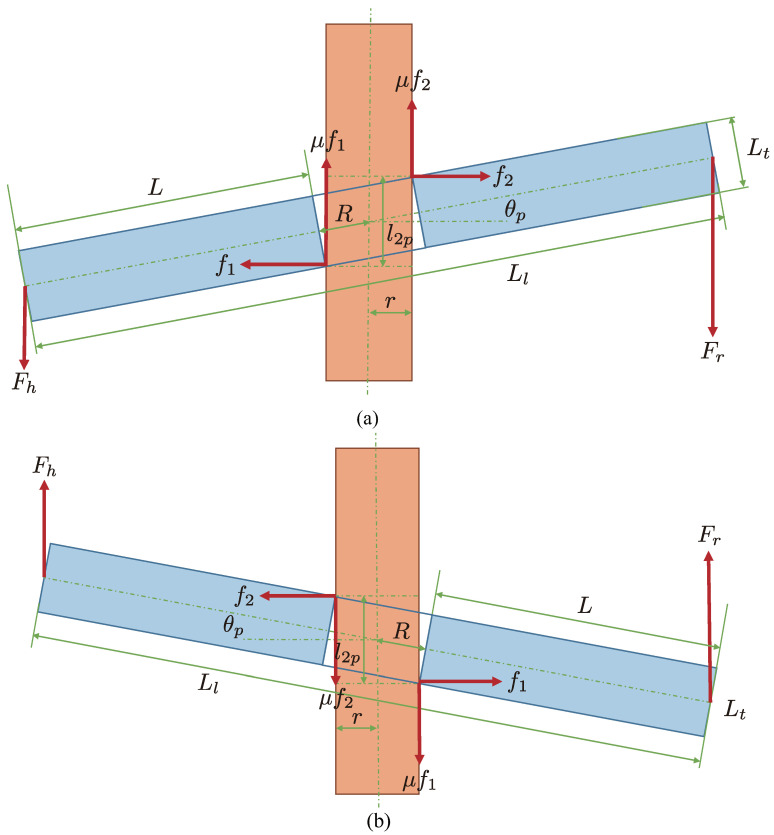
Human-Robot cooperative peg-in-hole task. In addition to the illustrated force, gravitational acceleration *g* always acts on the board. (**a**) in the case of insertion (downward motion); (**b**) in the case of removal (upward motion).

Next, we consider a condition in the case in which a lock phenomenon occurs between the peg and the hole in the board. The lock phenomenon means that a large contact force occurs between the peg and the hole, and the board cannot be moved in this state; i.e., the board is in a stationary state when the lock phenomenon occurs. Thus, let us consider force conditions from the viewpoints of the upward and downward directions and the rotational direction shown in Figure 17a. The force conditions can be described as follows:

(32) $$\begin{array}{ccc} & & mg+F_{h}+F_{r}\geq \mu f_{1}+\mu f_{2}\;, \end{array}$$

(33) $$\begin{array}{ccc} & & f_{1}=f_{2}\;, \end{array}$$

(34) $$\begin{array}{ccc} & & F_{r}L_{l}\cos\theta_{p}+\frac{l_{2p}}{2}\left(f_{1}+f_{2}\right)+r\mu f_{1}=F_{h}L_{l}\cos\theta_{p}+r\mu f_{2}, \end{array}$$

where

(35) $$\begin{array}{ccc} & & l_{2p}=\sqrt{4\left(R^{2}+r^{2}\right)+w^{2}}\;, \end{array}$$

(36) $$\begin{array}{ccc} & & L=\frac{L_{l}}{2}-R\;, \end{array}$$

(37) $$\begin{array}{ccc} & & \cos\theta_{p}=\frac{r}{R}. \end{array}$$

Deleting the forces $f_{1}$ and $f_{2}$ from Equations (32)–(34), the following equation can be obtained:

(38) $$mg\geq \left(\frac{2\mu L_{l}r}{l_{2p}R}-1\right)F_{h}-\left(1+\frac{2\mu L_{l}r}{l_{2p}R}\right)F_{r}.$$

When the above force condition is satisfied, the insertion can be executed without the lock phenomenon between the peg and the hole.

In addition, we consider the case of taking the board off of the peg. Since the friction force is the opposite to the case of the insertion, the signs of the terms $\mu f_{1}$ and $\mu f_{2}$ becomes the opposite (Figure 17b). Therefore, we can obtain

(39) $$mg\geq \left(-\frac{2\mu L_{l}r}{l_{2p}R}-1\right)F_{h}+\left(-1+\frac{2\mu L_{l}r}{l_{2p}R}\right)F_{r}.$$

In Equation (38), when the robot does not collaborate with human motion, the achievement of the task strongly depends on the sign of the scale $\left(\frac{2\mu L_{l}r}{l_{2p}R}-1\right)$. Actually, since the force $F_{h}$ will become negative, the insertion can be achieved even if the condition $\left(\frac{2\mu L_{l}r}{l_{2p}R}-1<0\right)$ is satisfied. On the other hand, in Equation (39), which means taking the board off of the peg, the force $F_{h}$ has to be a positive value. This means that the task cannot be achieved without robot collaboration.

#### 6.1.3. Posture Condition

In addition to the force condition, the posture (pitch angle) condition of the board is also considered in the collaborative peg-in-hole task as a stringent condition. From the radii *r* and *R* and the thickness $L_{t}$, the posture condition can be obtained as follows:

(40) $$\begin{array}{ccc} 2r & < & 2R-2L_{t}\sin\theta_{p} \end{array}$$

(41) $$\begin{array}{ccc} \frac{R-r}{L_{t}} & > & \sin\theta_{p} \end{array}$$

(42) $$\begin{array}{ccc} \theta_{p} & < & \sin^{-1}\left(\frac{R-r}{L_{t}}\right) \end{array}$$

If this condition is satisfied during the collaborative motion, the collaborative peg-in-hole task can be achieved without satisfying the force condition, because contact between the peg and the hole does not occur. This condition can be satisfied by our real-time Human-Robot collaborative system in limited circumstances.

### 6.2. Experimental Result

We show one application of the Human-Robot collaborative system; that is, a peg-in-hole task carried out by a human and a robot. In the experiment, the radii (R,r) of the peg and the hole were 6.350 mm and 6.325 mm, respectively. Thus, since the clearance was only 0.025 mm (25 μm), precise motion and positioning were essential. Moreover, since it is very difficult to achieve the peg-in-hole task successfully, this task was considered to be valid for the verification of the effectiveness of our high-speed Human-Robot collaborative system.

Table 2 shows the experimental parameters of the collaborative peg-in-hole task. Substituting the experimental parameters into the above conditions for achieving the collaborative peg-in-hole task (Equations (30) and (31)), the following condition can be obtained:

(43) $$\left|e_{i}^{\prime }\right|\leq w+cR=1+0.025\approx 1.13\left[\mathrm{mm}\right]$$

(44) $$\theta_{m}=\cos^{-1}\left(\frac{r}{R}\right)=\cos^{-1}\left(\frac{6.325}{6.35}\right)\approx 8.88\times 10^{-2}\left[\mathrm{rad}\right]\approx 5.09\left[{}^{\circ}\right]$$

(45) $$\theta_{p}<\sin^{-1}\left(\frac{R-r}{L_{t}}\right)=\sin^{-1}\left(\frac{6.35-6.325}{5}\right)\approx 5.00\times 10^{-3}\left[\mathrm{rad}\right]\approx 2.87\times 10^{-1}\left[{}^{\circ}\right]$$

If the conditions $e_{i}<e_{i}^{\prime }$ and $\theta^{\prime }<\theta_{m}$ are satisfied, we can achieve the collaborative peg-in-hole task. Since the posture condition shown in Equation (42) is sufficient for achieving the collaborative peg-in-hole task, we do not necessarily need to satisfy this condition.

In addition, in order to confirm the validity of the force condition, we conducted a preliminary experiment in which the board into which the peg was inserted was pulled off of the peg by a human subject. As a result, the lock phenomenon occurred, and the human subject could not move the board. This means that the force condition was not satisfied.

**Figure 18:**
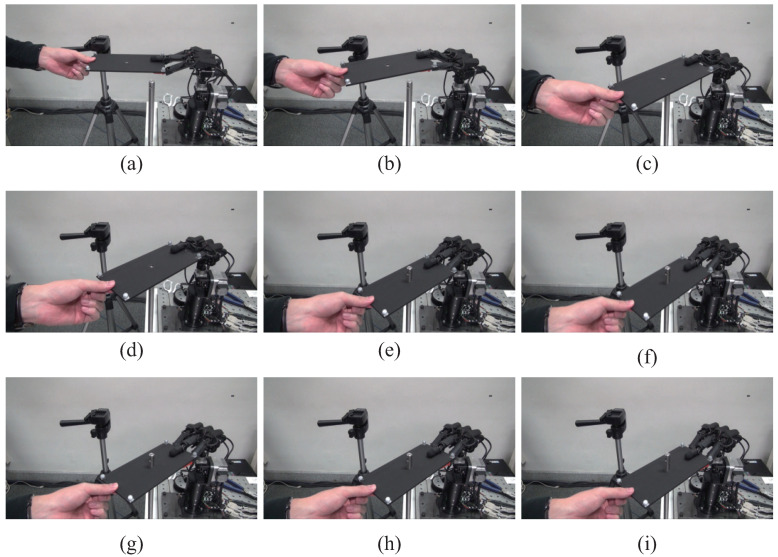
Collaborative peg-in-hole task. (**a**–**d**): collaborative motion; (**e**–**i**): collaborative peg-in-hole.

**Figure 19:**
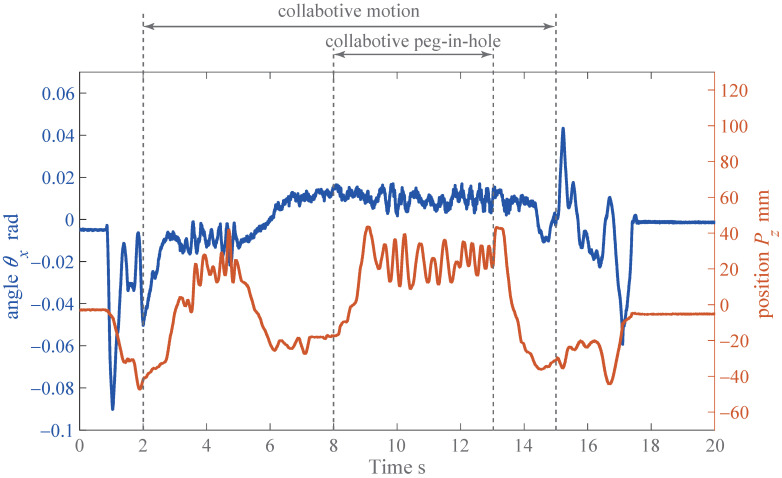
Data of collaborative peg-in-hole task.

Figure 18 and Figure 19 show the experimental results and data of the collaborative peg-in-hole task. Furthermore, a video of the experimental result is available on our website [32,33]. Figure 18a is the initial state. Figure 18a–d shows the collaborative motion, and Figure 18e–i shows the collaborative peg-in-hole task. From the experimental results, the collaborative peg-in-hole task was carried out successfully. In particular, a human could move the board upward and downward smoothly, even when the peg was inserted in the hole. In general, it was not possible to move the board smoothly when the peg was in the hole.

In Figure 19, the collaborative motion was performed in the period of 2–15 s, and the collaborative peg-in-hole task was also performed in the period of 8–13 s. From Figure 19, it can be seen that the board could be moved upward and downward without the lock phenomenon using our proposed method and developed system, even when the peg-in-hole task was achieved. In fact, even when the *z*-location $P_{z}$ of the board was varied upward and downward, the pitch angle $\theta_{x}$ was settled at around 0.1 rad. In addition, since the pitch angle $\theta_{x}$ was not varied instantly comparing with the *z*-location, we found that the insertion and removal actions could be achieved smoothly.

## 7. Conclusions

In this paper, we developed a high-speed, high-accuracy Human-Robot collaborative system using a high-speed robot hand and a high-speed camera. Furthermore, we proposed visual sensing and robot hand control methods that could run at 1000 Hz. Then, we analyzed the stability of the collaborative system and the collaborative error. We demonstrated collaborative motion and evaluated the collaborative error based on the analysis results and the control performance with various frame rates. As a result, we found that high-speed performance was critically important in the HRI system from the viewpoints of the collaborative error, system stability and control performance of the robot. Moreover, we tried to achieve a collaborative peg-in-hole task using the developed system. To achieve the task, we analyzed the condition for performing the collaborative peg-in-hole from the viewpoints of geometric, force and posture conditions. Finally, we demonstrated the collaborative peg-in-hole task successfully. As a result, the validity of the developed high-speed and high-accuracy collaborative system was confirmed.

In the future, using our collaborative system, we plan to demonstrate other tasks that cannot be achieved with human-human collaboration or conventional Human-Robot collaboration. Moreover, since user feedback contributes to improving the performance of the robot action during Human-Robot collaboration [36], we plan to develop more flexible and intelligent HRI systems during the interaction between humans and robots.

## Figures and Tables

**Table 1:** Experimental conditions.

Parameter	Value
$e_{r}$	0.21 pixel
$e_{p}$	0.1 pixel
*a*	7 μm/pixel
*f*	5 mm
$L_{c}$	800 mm
$L_{l}$	220 mm
*A*	50 mm
$f_{freq}$	5 Hz

**Table 2:** Experimental parameters for the collaborative peg-in-hole task.

Parameter	Value
*R*	6.350 mm
*r*	6.325 mm
β	45°
*w*	1 mm
$L_{t}$	5 mm
$L_{l}$	100 mm
